# The human acetylcholinesterase C-terminal T30 peptide activates neuronal growth through alpha 7 nicotinic acetylcholine receptors and the mTOR pathway

**DOI:** 10.1038/s41598-023-38637-1

**Published:** 2023-07-15

**Authors:** Alexandru Graur, Patricia Sinclair, Amanda K. Schneeweis, Daniel T. Pak, Nadine Kabbani

**Affiliations:** 1 School of Systems Biology, George Mason University, 4400 University Drive, Fairfax, VA 22030 USA; 2 Interdiscplinary Program in Neuroscience, George Mason University, Fairfax, VA 22030 USA; 3 Department of Pharmacology and Physiology, Georgetown University Medical Center, Washington, DC USA

**Keywords:** Neuroscience, Cellular neuroscience, Molecular neuroscience, Cell growth, Cell signalling

## Abstract

Acetylcholinesterase (AChE) is a highly conserved enzyme responsible for the regulation of acetylcholine signaling within the brain and periphery. AChE has also been shown to participate in non-enzymatic activity and contribute to cellular development and aging. In particular, enzymatic cleavage of the synaptic AChE isoform, AChE-T, is shown to generate a bioactive T30 peptide that binds to the ⍺7 nicotinic acetylcholine receptor (nAChR) at synapses. Here, we explore intracellular mechanisms of T30 signaling within the human cholinergic neural cell line SH-SY5Y using high performance liquid chromatography (HPLC) coupled to electrospray ionization mass spectrometry (ESI–MS/MS). Proteomic analysis of cells exposed to (100 nM) T30 for 3-days reveals significant changes within proteins important for cell growth. Specifically, bioinformatic analysis identifies proteins that converge onto the mammalian target of rapamycin (mTOR) pathway signaling. Functional experiments confirm that T30 regulates neural cell growth via mTOR signaling and ⍺7 nAChR activation. T30 was found promote mTORC1 pro-growth signaling through an increase in phosphorylated elF4E and S6K1, and a decrease in the autophagy LC3B-II protein. These findings are corroborated in hippocampal neurons and show that T30 promotes dendritic arborization. Taken together, our findings define mTOR as a novel pathway activated by T30 interaction with the nAChR and suggest a role for this process in human disease.

## Introduction

Acetylcholine (ACh) is an abundant neurotransmitter in the brain and periphery important for various physiological functions including movement, memory, and immune system regulation^1^. The cholinergic synapse is among the most well understood synapses within many organisms, serving as a prototype for classical neurotransmission^2,3^. Amongst the primary molecular components of the cholinergic synapse are ACh binding receptors such as the ligand-gated nicotinic acetylcholine receptor channel (nAChR)^4^. In addition to their post-synaptic localization, nAChRs are also found presynaptically and can contribute to synaptic growth and neurotransmitter release in brain circuits for memory and cognitive processing^5^. The α7 nAChR is a widespread homopentameric channel receptor that activates calcium within cells^6^. Studies show that α7 nAChRs can signal through both ionotropic and metabotropic modes in neural and immune cells^7^. In particular, α7 nAChR signaling is important for neural cell development and synaptic growth^8–10^.

**Figure 1:**
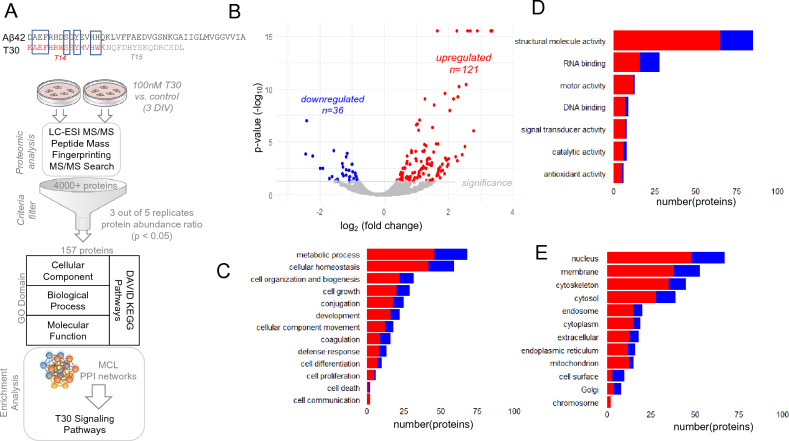
Proteomic analysis of T30 signaling. (**A**) Top: The T30 peptide sequence contains active (T14) and inactive (T15) portions as well as some homology to Aβ42. Bottom: Cells were treated with 100 nM T30 for 3 DIV then analyzed by LC–ESI MS/MS and bioinformatics. (**B**) The distribution of detected proteins within T30 treated cells. The threshold for statistical significance (p < 0.05). (**C**–**E**) Gene Ontology (GO) terms associated with significantly altered proteins (**C** biological process, **D** molecular function. **E** Cellular component).

The cholinergic synapse is marked by the presence of acetylcholinesterase (AChE), a powerful enzyme that regulates ACh levels within the synaptic cleft^11^. AChE however is also a well-established signaling molecule with hydrolytic and non-hydrolytic functions including strong trophic activity^12–14^. The mammalian AChE gene contains six exons which are spliced in several alternative forms that create three main AChE isoforms (AChE-T,-R, -H)^11,14^. The synaptic tetrameric variant AChE-T is the dominant isoform in the brain^14^. AChE-T is a cell membrane attached enzyme via its well characterized proline-rich membrane anchor (PRiMA) domain^11,11,14^. AChE-T also has an amphiphilic region within its c-terminus that contributes to oligomerization^15^. Proteolytic cleavage of the last 30 amino acids at the c-terminus generates in vitro, as well as in vivo a T30 bioactive peptide^16,17^. Interestingly, since the c-terminal region of AChE-T contains some sequence homology with the amyloid precursor protein (APP), cleavage of AChE-T and APP appears to yield two peptides (T30 and Aβ42, respectively) with some sequence similarity (Fig. 1A) and neurotoxic potential^17^.

Interestingly Aβ42 and T30 are reported to both bind to the ⍺7 nAChR, and can impact calcium signaling and neurotransmission^18–21^. The pharmacological targeting of T30 is a promising approach for neurodegenerative and amyloid related disease^22^. In this study we explore how T30 impacts signaling within the human neural cholinergic cell line SH-SY5Y, which is a model for Alzheimer’s Disease (AD) and known to endogenously express ⍺7 nAChRs^23,24^. Using quantitative whole cell proteomics and targeted functional cell assays, we identify a novel mechanism of T30 signaling via mTOR that leads to cell growth. T30 activation of mTOR may provide a mechanistic understanding of how non-enzymatic AChE-T can contribute to synaptic function.

## Methods

### Cell culture, transfection, and treatment

Human neuroblastoma cells SH-SY5Y cells (ATCC CRL-2266) were cultured in T75 flasks for propagation and then plated onto 100 μg/ml polyD-lysine (Millipore, A-003-E) coated 96-well glass bottom plates for imaging. Cells were fed DMEM (Gibco 11995065) supplemented with 10% fetal bovine serum (FBS) and 1% pen/strep at 37 °C and 5% CO_2_. Experiments were conducted in cells that did not exceed 19 passages. For treatment experiments, cells were grown to 70% confluence then treated with: 100 nM T30/T15/ NBP14, 50 nM α-bungarotoxin (bgtx) (Thermo Fisher B1601), 1 μM Rapamycin (Thermo Scientific AAJ62473MF). Treatment media was changed daily. T30, T15, and NBP14 peptides were provided by NeuroBio LTD (Oxford, UK) and have been characterized elsewhere^20,25,26^. Sequences for these peptides are listed in Supplemental Table S1. Cells were transfected using Lipofectamine 2000 (Thermo Fisher 11668030) with constructs encoding α7_345–348A_ in pEYFP-C1^27^ with the pEYFP-C1 plasmid used as a control. All cDNA constructs were propagated in DH5α cells (Thermo Fisher 18258012) and purified using a maxi prep kit (Xymo Research, Irvine, CA, USA). Cell proliferation was analyzed through live cell counting using phase contrast microscopy within a C-Chip hemocytometer (Bulldog Bio, Portsmouth, NH, USA).

For primary cultures, a total of 2 pregnant Sprague–Dawley rat mothers (8–10-week-old females, RRID: RGD_734476) were obtained from Charles River (Raleigh, NC) with all animal experimental procedures approved in accordance with guidelines and regulations of the Georgetown University Institutional Animal Care and Use Committee and ARRIVE guidelines. All efforts were made to minimize distress during euthanasia. At embryonic day 19 (E19), pregnant rats were euthanized using a flow-regulated carbon dioxide chamber, and death was verified by toe pinch and decapitation. Anesthetics were not used prior to euthanasia due to interference of proper neuronal growth in hippocampal neuronal cultures. Primary hippocampal neurons were obtained from E18 Sprague–Dawley rats and cultured onto poly-d-Lysine (Sigma cat# P0899-1G; 100 µg/m) and laminin (Sigma-Aldrich L2020; ~ 6.25 µg/mL) coated coverslips at 75,000 cells/well as previously described^28^. Cells were grown in a Neurobasal media with SM1 (StemCell cat# 05711), 12.5 μM glutamate, 500 μM glutamine, and 0.1 mg/mL Primocin (InvitroGen cat# ant-pm-1)] at 37 °C/5% CO_2_. At 2 DIV (days in vitro) cells were treated with 1 nM, 100 nM, 1 μM T30 or the vehicle (H_2_O) added to the culture media.

### Protein extraction and western blot

Proteins were obtained from cultured cells as previously described^29^. In brief, at 3 DIV cells were lysed using a 0.1% Triton X-100 lysis buffer (Triton X-100, 150 mM NaCl, 20 mM Tris HCl, 2 mM EDTA, and 10% glycerol) supplemented with protease (Complete Mini, Roche) and PhosSTOP (Sigma Aldrich 4906845001) inhibitors. Protein concentration was determined using the Bradford assay. Proteins were separated on a NuPAGE 4–12% Bis–Tris gradient gel (Thermo Fisher NP0322BOX) and then transferred onto a nitrocellulose membrane (Thermo Fisher IB301002). Membranes were blocked with milk prior to application of a primary antibody: GAPDH (1:1000 Cell Signaling 5174), LC3B (1:1000 Cell Signaling 2775), p-eIF4E (1:1000 Cell Signaling 9741), eIF4E (1:1000 Santa Cruz sc-271480), p-S6K1 (Thr389) (1:1000 Cell Signaling 9205), S6K1 (1:1000 Santa Cruz sc-8418) and Cytochrome C (1:1000 AbCam ab90529). HRP secondary antibodies were purchased from Jackson Immunoresearch (West Grove PA, USA). A SeeBlue Plus2 Ladder (Thermo Fisher LC5925) was used as molecular weight marker. Bands were visualized using SuperSignal West Pico or SuperSignal West Femto Chemiluminescent substrates (Thermo Fisher) via the G:BOX Imaging System and GeneSYS software (Syngene, Fredrick MD, USA). Band density was analyzed in Image J (NIH, Bethesda MD, USA). All measures were normalized to GAPDH unless otherwise stated. Average band intensity measures are based on three separate experiments.

### Liquid-chromatography electrospray ionization mass spectrometry

Whole cell proteomic analysis was performed based on an established method^19,30^. Briefly, solubilized protein samples were incubated for 5 min with acetone on ice followed by protein precipitation via centrifugation. The resulting protein pellet was denatured, reduced, and alkylated with 8 M urea, 1 M dithiothreitol, and 0.5 M iodoacetamide. Proteins were digested with trypsin (0.5 μg/μl) in 500 nM ammonium bicarbonate and incubated at 37 °C for 5 h. The samples were then desalted with C-18 ZipTips (Millipore), dehydrated in a SpeedVac for 18 min and reconstituted in 0.1% formic acid before undergoing liquid-chromatography electrospray ionization mass spectrometry (LC-ESI MS/MS) with 5 technical replicates.

LC-ESI MS/MS was performed using an Exploris Orbitrap 480 equipped with an EASY-nLC 1200HPLC system (Thermo Fischer Scientific, Waltham, MA, USA). Peptides were separated using a reverse-phase PepMap RSLC 75 μm i.d by 15 cm long with a 2 μm particle size C18 LC column (Thermo Fisher Scientific, Waltham, MA, USA), and eluted with 80% acetonitrile and 0.1% formic acid at a flow rate of 300 nl/min. After a full scan at 60,000 resolving power from 300 to 1200 m/z, peptides were fragmented by high-energy collision dissociation (HCD) with a normalized collision energy of 28%. EASY-IC filters for monoisotopic precursor selection, internal mass calibration, and dynamic exclusions (20 s) were enabled. Data on peptide precursor ions with charge states from + 2 to + 4 was recorded.

### Proteomic quantification and statistical analysis

The SEQUEST HT search engine within the Proteome Discoverer *v*2.4 (Thermo Fisher Scientific, Waltham, MA, USA) was used to identify proteins by comparing raw MS peptide spectra to the NCBI 2018 human protein database using the following search engine parameters: mass tolerance for precursor ions = 2 ppm; mass tolerance for fragment ions = 0.05 Da; and cut-off value for the false discovery rate (FDR) in reporting peptide spectrum matches (PSM) to the database = 1%. Peptide abundance ratios were determined by precursor ion quantification in Proteome Discoverer *v*2.4, with the vehicle control group used as the denominator. Statistically significant abundance ratios with adjusted p-values < 0.05 were determined using a one-way analysis of variance (ANOVA) followed by Benjamini–Hochberg post-hoc analyses. Proteins with a quantifiable spectra signal profile seen in at least 3 of the 5 technical replicates were included in the analysis. Markov Cluster Algorithm (MCL) with an inflation parameter of 3 was used to perform clustering analysis on the data in the STRING database. Data was analyzed, organized, and presented using the R package (R Core Team, 2021): ggplot2^31^, tidyverse^32^, Excel, the Database for Annotation, Visualization, and Integrated Discovery (DAVID) and Search Tool for the Retrieval of Interacting Genes/Proteins (STRING, *v*11.5) application^33^.

### Immunocytochemistry and cell imaging

SH-SY5Y cells were fixed in a solution consisting of 1 × PEM (80 mM PIPES, 5 mM EGTA, and 1 mM MgCl2, pH 6.8) and 0.3% glutaraldehyde then quenched with sodium borohydride (2 mg/ml). Cells were permeabilized using 0.05% Triton X-100 (Sigma Aldrich). Quantification of structural change (i.e., neurite shape and growth) was performed using rhodamine phalloidin (Cytoskeleton PHDG1-A). All morphometric measures were conducted and quantified using ImageJ (NIH, Bethesda, MD, USA) as described^8^. α7 nAChRs were detected at the cell surface and within the cytoplasm using Alexa Fluor 488 conjugated α-bungarotoxin (Alexa-488 bgtx) (Thermo Fisher B13422) as described^27^. Images were captured using an inverted Zeiss LSM800 confocal microscope and the Zen software package (Carl Zeiss AG, Oberkochen, Germany).

Primary culture coverslips were fixed at 7 DIV with 1% paraformaldehyde and 4% sucrose at room temperature for 7 min then submerged in methanol for 7 min at − 20 °C. Cells were immunolabeled with antibodies to MAP2 (PhosphoSolutions, cat# 110-MAP2) in a GDB solution consisting of 30 mM phosphate buffer (pH 7.4) containing 0.1% gelatin, 0.3% Triton X-100, and 450 mM NaCl at 4 °C overnight. Species specific secondary antibodies (AlexaFluor 488 (Invitrogen) were applied in GDB for 2 h at 25 °C, then washed, and coverslips mounted in NPG (glycerol, 1 M Tris pH 8, 0.05 g n-propyl gallate). Analysis of dendritic branching by Sholl analysis was performed on ImageJ v1.53t (https://imagej.net/software/fiji/downloads) via the Neuroanatomy plugin^34,35^.

## Results

### Identification of a T30 reactive proteome within neural cells

The human neuroblastoma SH-SY5Y cell line is a well-established model for the study of neural cell development and neurodegeneration^24,36^. SH-SY5Y cells maintain the ability to model cholinergic neurons with endogenous expression of various cholinergic receptors^23,37^. We used SH-SY5Y cells to examine proteomic responses to treatment with the bioactive c-terminal peptide of the AChE-T enzyme T30^25^. Cells were treated with 100 nM T30 for 3 DIV then processed for proteomic analysis. T30 has been identified as an endogenous ligand of the ⍺7 nAChR, and at 100 nM it is shown to activate ⍺7 nAChR calcium signaling in various cell lines^25,38^. In these experiments, we used the vehicle treatment condition as the control group in protein comparison.

We have optimized a shotgun LC/ESI–MS/MS approach to identify cellular proteins, for SH-SY5Y and other cell lines, in response to various stimuli^19,30^. In this study, LC/ESI–MS/MS peptide detection was used to quantify protein changes based on label-free precursor ion abundance ratio measures between T30 treated cells and control samples (Fig. 1A). Our proteomic analysis identified 4331 soluble cellular proteins in the sample. A volcano plot distribution (Fig. 1B) shows that 121 of these proteins were significantly increased while 40 were significantly decreased and 4170 did not statistically change between the two experimental conditions (p < 0.05). Using Proteome Discoverer, we annotated the significantly altered proteins according to the three main GO domains: biological processes, molecular function, and cellular components. GO domain terms matching the greatest number of altered proteins in response to T30 treatment are presented in Fig. 1C–E. A full list of the significantly altered proteins and their corresponding peptide scores is provided Supplemental Table S2.

**Figure 2:**
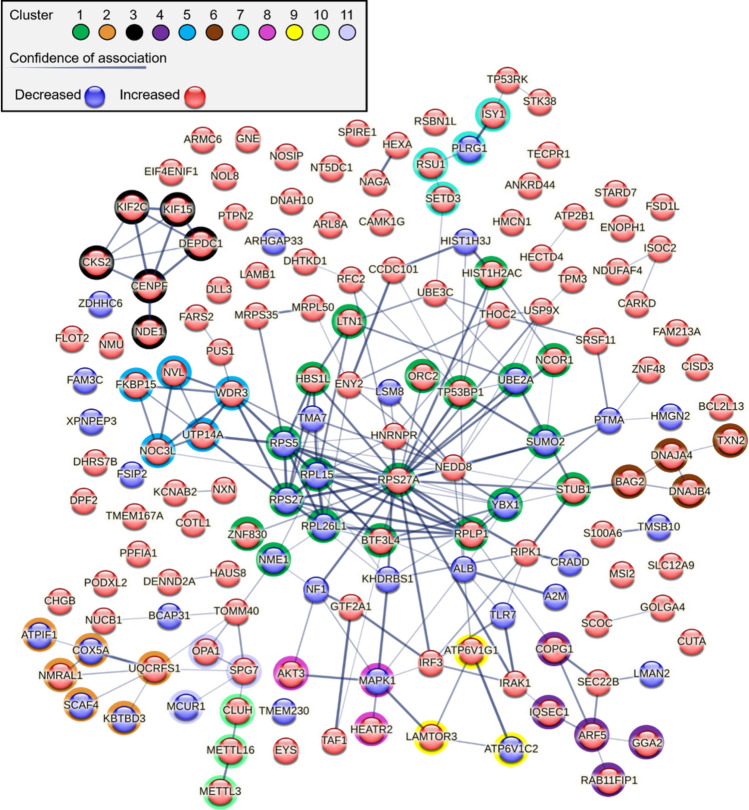
STRING network and cluster analysis of the T30 proteome. STRING analysis of the T30 proteome (consisting of all significantly altered proteins) showing networks for interacting proteins. Line thickness reflects the confidence between node associations, and node color indicates whether the protein is increased or decreased. A Markov cluster algorithm (MCL) was used to identify 11 functional clusters within the proteome network.

Whole cell proteomics enables an analysis of functional changes within cells that can be supported by further examining protein–protein interaction (PPI) networks^39^. Modifications to PPI networks can reveal important information on functional adaptive responses to an extracellular signal. We used a Markov cluster (MCL) analysis to define PPI networks within the T30 proteome (consisting of all significantly upregulated and downregulated proteins)^41^. MCL analysis shows a relatively integrated PPI network based on the identity of the significantly altered proteins (Fig. 2). Within this PPI network we identified several altered protein clusters and impacted cell pathways. The largest PPI cluster was found to contain 19 proteins with 53 connections yielding a significant PPI enrichment (p < 1.11 × 10^–16^). This cluster (Cluster 1) consisted of an average local clustering coefficient (ALCC) of 0.697. Enrichment analysis of Cluster 1 confirms ribosome enrichment in KEGG Pathways with a false discovery rate (FDR) of 8.49 × 10^–7^. MCL analysis revealed 11 clusters within the T30-associated PPI network (Fig. 2 and Table 1). Many of the identified clusters were involved in pathways for cell growth and protein regulation.

**Table 1:** Top clusters identified using MCL in STRING.

Cluster #	Node #	Edge #	ALCC	PPIE	Cluster enrichment
1	19	53	0.697	1.11E−16	Ribosome
2	6	6	0.75	6.95E−09	Electron transport chain/mitochondria
3	6	11	0.933	5.99E−13	Microtubule binding
4	5	4	0.8	2.71E−07	Protein transport
5	5	9	0.9	5.71E−10	Ribosome biogenesis in eukaryotes
6	4	4	0.833	1.26E−07	Chaperone
7	4	3	0.5	1.78E−05	Spliceosome
8	3	2	0.667	8.47E−03	mTOR signaling pathway
9	3	3	1	1.16E−06	MTORC1 regulation
10	3	2	0.667	4.33E−05	RNA binding
11	3	2	0.667	8.58E−05	Mitochondrial calcium ion transmembrane transport

### T30 activates an mTOR pathway for cell growth

**Figure 3:**
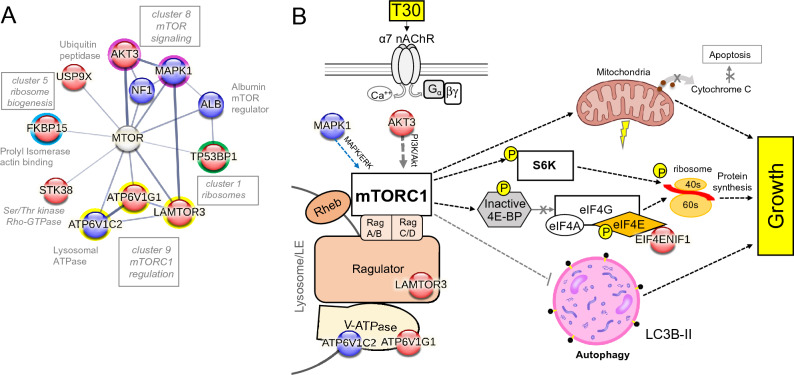
Identification of the mTOR pathway as a cellular target of T30 action. (**A**) mTOR is found at the convergence for several proteins and MCL clusters (1, 5, 7, 9). (**B**) A signal transduction hypothesis model for T30 activation of mTOR in cells. Proteins discovered through the proteomic assay are indicated within the red (increased) and blue (decreased) circles.

Studies have shown a role for non-hydrolytic AChE function in neuronal growth and synaptic maturation^13,42^. In particular, the c-terminal fragment T30 that is produced by AChE-T cleavage has been shown to activate intracellular signaling important for neural development^43,44^. Our proteomic analysis reveals new intracellular proteins and signaling pathways that are altered in response to a 3-day T30 presentation within the SH-SY5Y cell line. Bioinformatic KEGG Pathway analysis in DAVID further revealed enrichment of proteins involved in mTOR signaling. Our analysis indicates that mTOR may serve as a point of convergence between PPI networks and several of the identified clusters within the dataset (Fig. 3A). In Fig. 3B, a mechanistic model summarizing the hypothesized involvement of mTOR in T30 mediated growth is presented. In this model, differentially altered (increased and decreased) proteins identified within the proteome are important components of mTOR signaling. The proteomic data also suggests that T30 promotes mTORC1 signaling through the regulation of downstream proteins.

**Figure 4:**
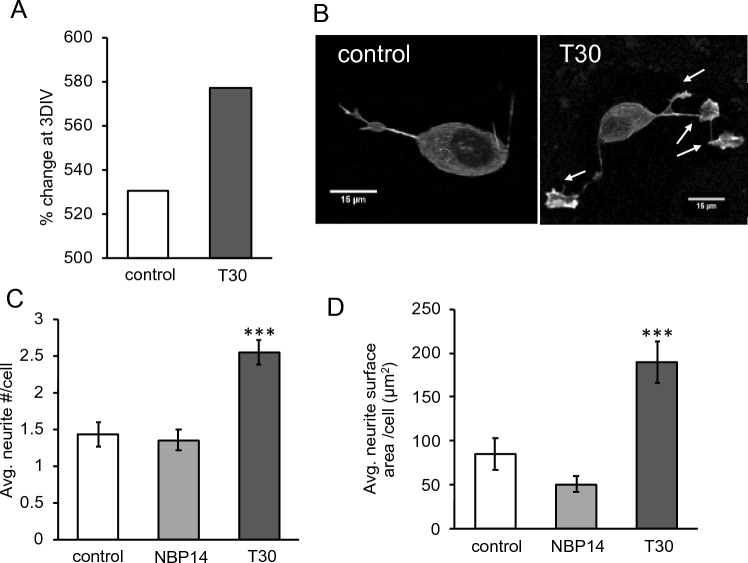
T30 treatment regulates neural growth. SH-SY5Y cells were treated with 100 nM T30 for 3 days in vitro (DIV) then imaged using fluorescent (f-actin) phalloidin. (**A**) Cell proliferation shown as a percentage change in total cell number after 3 DIV. (**B**) Representative cell images at 3 DIV. Arrows point to filipodia and lamellipodia structures. (**C**) Average neurite number per cell. (**D**) Average neurite surface area per cell. n = 40, ***p < 0.001.

To test this model, we examined SH-SY5Y cell growth in the presence of the 100 nM T30 for 3 DIV. As shown in Fig. 4A, T30 treatment increased cell proliferation but this effect was not found to be statistically significant (p = 0.287). Protruding from the membrane of developing neural cells are motile structures that consist of actin projecting lamellipodia as well as cytoplasmic filopodia^45,46^. In previous studies we have shown a role for ⍺7 nAChRs in regulating actin-mediated cytoskeletal growth in neurites and growth cones^8,29^. T30 presentation showed a significant increase in neurite growth as measured by neurite number, increased presence of filipodia/lamellipodia structures, and total measured surface area (n = 40, p < 0.001). This effect was not seen in response to the application of a cyclic variant of the T30 peptide termed NBP14 that is shown to be biologically inert^47^ (n = 40, p = 0.369) (Fig. 4B–D).

### A requirement for ⍺7 nAChR signaling in T30-mediated growth

**Figure 5:**
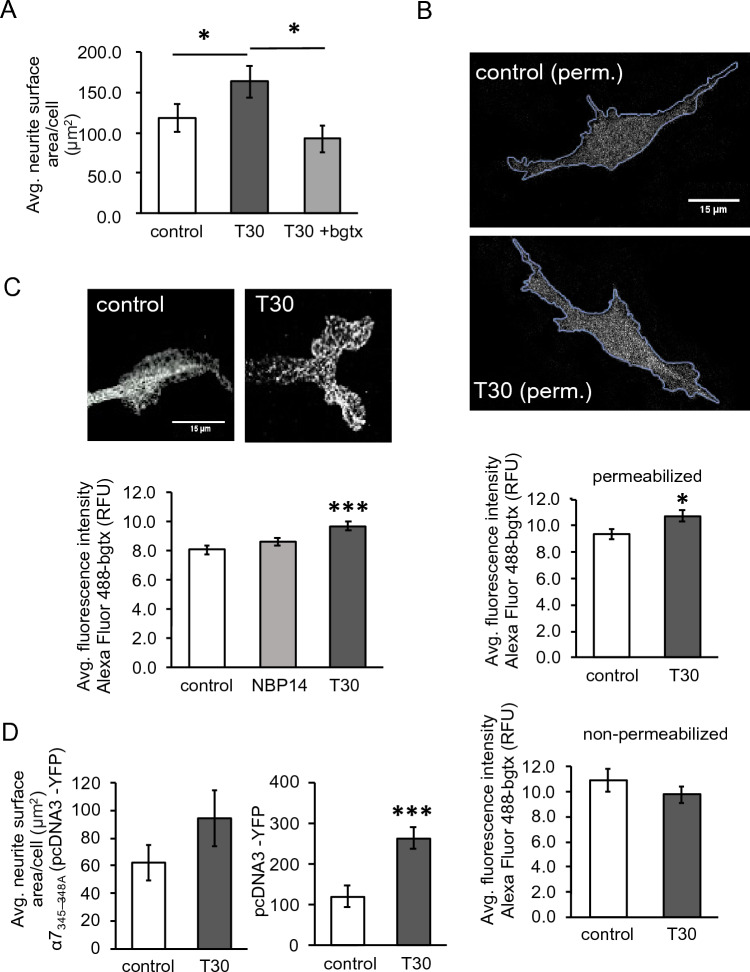
T30 activates ⍺7 nAChR signaling to promote neural cell growth. (**A**) Average neurite surface area at 3 DIV. (**B**) Images, representative permeabilized cell images showing ROI analysis of Alexa-488-bgtx signal intensity. Histograms, average fluorescence intensity of the Alexa-488-bgtx signal in permeabilized and non-permeabilized cells. (**C**) Average fluorescence intensity of the Alexa-488-bgtx signal within sites of growth. (**D**) Average neurite surface area within cells transfected with α7_345–348A_ or pEYFP-C1. n = 40, *p < 0.05, ***p < 0.001.

Studies show that T30 binds to ⍺7 nAChRs in neural cells activating intracellular calcium signaling and increasing nAChR expression^48^. We confirmed the role of ⍺7 nAChRs in T30-mediated neurite growth using the selective ⍺7 nAChR blocker ⍺-bungarotoxin (bgtx). As shown in Fig. 5A, co-treatment of cells with 50 nM bgtx and T30 did not produce an effect on growth (n = 40, p = 0.136). We examined the impact of T30 treatment on the expression and localization of the ⍺7 nAChR within SH-SY5Y cells. Fluorescence imaging was performed using Alexa 488-bgtx to assess ⍺7 nAChR expression as previously shown^27^. We first compared cell surface to intracellular expression by labeling cells with Alexa 488-bgtx in  non-permeabilized and permeabilized fixation protocols, respectively. Data shows that T30 increases the Alexa 488-bgtx signal within the cell (n = 40, p < 0.05) but not at the cell surface (n = 40, p = 0.139) (Fig. 5B). We examined the effect of T30 on ⍺7 nAChR expression at subcellular sites of growth. Our previous findings show that this nAChR is targeted to the growth cone and can regulate neurite motility through the cytoskeleton^8,49^. As shown in Fig. 5C, treatment with T30 was found to significantly increase the Alexa 488-bgtx signal at growth sites relative to both the control condition (n = 30, p = 0.0002) as well as the cyclic NBP14 peptide (n = 30, p = 0.005).

The activation of the α7 nAChR can regulate neurite development within hippocampal neurons through the ability of the α7 nAChR to bind and activate heterotrimeric GTP-binding proteins (G proteins)^27^. Expression of a mutant α7 subunit (α7_345–348A_) that lacks the G protein-binding site has been established as a method for blocking α7 nAChR-mediated G protein signaling and diminishing the receptor's metabotropic effect on growth^27^. We tested the ability of T30 to promote growth in SH-SY5Y cells transfected with α7_345–348A_ (pcDNA3-YFP). In this assay, control cells were transfected with the vector pcDNA3-YFP alone. Morphological analysis shows that T30 treatment does not increase neurite growth in cells expressing α7_345–348A_ (n = 40, p = 0.09) (Fig. 5D).

### T30 activates the mTOR1 pathway

**Figure 6:**
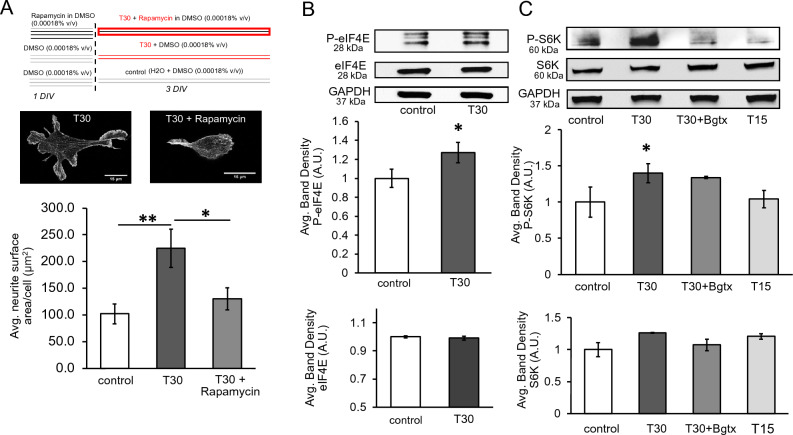
Analysis of the mTOR pathway during T30 mediated growth. (**A**) Top, summary of the rapamycin experiment. Middle images, representative cells at 3 DIV. Bottom histogram, average neurite surface area measures. (**B**,**C**) Top, representative immunoblots. Bottom, average band density measures from 3 separate experiments. n = 40, *p < 0.05, **p < 0.005.

Our proteomic analysis reveals an enrichment of intracellular proteins involved in mTOR signaling (Fig. 3 and Table 1). Activation of mTOR is a conserved evolutionary signaling strategy for balancing cell growth and metabolic demand in various context^50–52^. Previous work indicates that α7 nAChRs engage mTOR during development, inflammation, and cancer progression^53^. We directly confirmed the involvement of mTOR signaling in T30-mediated neurite growth using the mTOR inhibitor rapamycin. As shown in Fig. 6A, pre-treatment of neural cells with 1uM rapamycin, for 24 h, was found sufficient to abolish the effect of T30 on neurite growth (n = 40, p = 0.161). The activation of mTORC1 is shown to promote the phosphorylation of the translation regulating factor eIF4E (serine 209) and the ribosomal protein S6 kinase (S6K) (threonine 389) during mTOR-mediated translational regulation and cell growth^54,55^ (Fig. 3B). We assessed the effect of T30 on eIF4E and S6K expression and phosphorylation at 3 DIV. As shown in Fig. 6B,C, T30 treatment did not increase the expression of eIF4E nor S6K but increased their phosphorylation (p = 0.011and p = 0.03, respectively).

**Figure 7:**
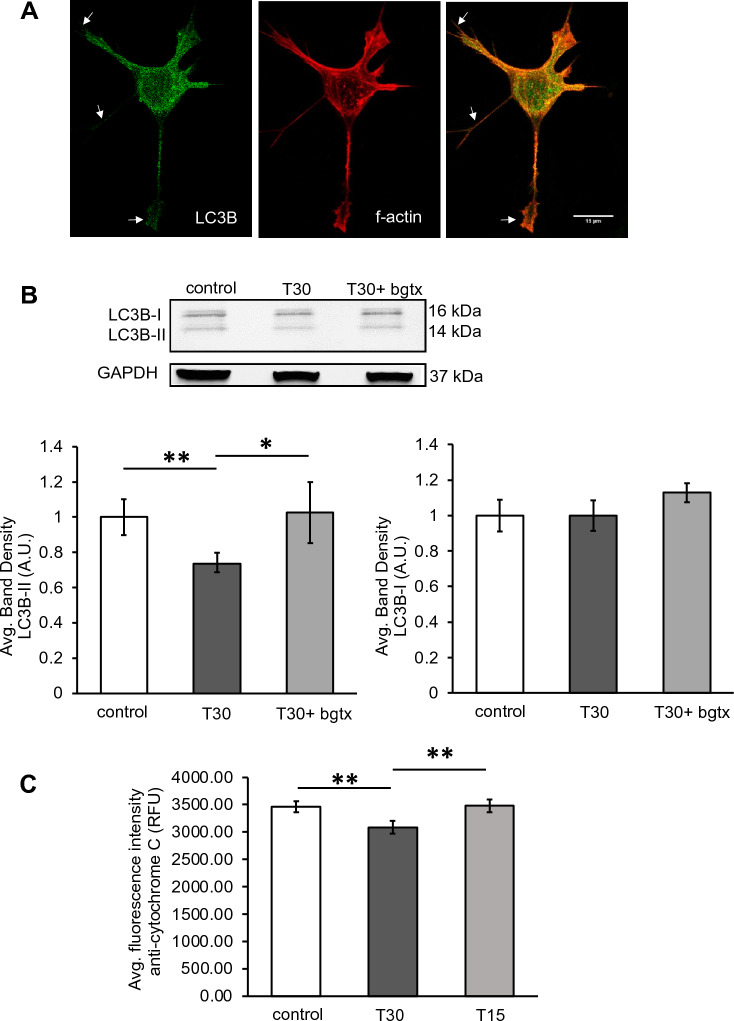
T30 treatment is associated with a reduction in autophagy markers and cytochrome c levels. (**A**) Representative cell images showing T30 treatment at 3DIV. Arrows point to the localization of LC3B in the growing neurite. (**B**) Top, representative immunoblots. Bottom, average band density measures. (**C**) Average fluorescence intensity measure of the anti-cytochrome c immunosignal. n = 40, * p < 0.05, ** p < 0.005.

The mTOR pathway plays a critical role in maintaining cellular balance between anabolic and catabolic states through the regulation of degradation-mediated autophagy processes^56^. Isoforms of the cytosolic light chain (LC3) protein undergo modifications during autophagy and thus serve as autophagy markers^57^. We examined autophagy-associated  LC3I to II conversion within LC3B as previously shown^57^. LC3B was detected throughout the cell, including sites of growth, consistent with the role of the autophagosome in modulating structural growth^58^ (Fig. 7A). Treatment of cells with T30 (for 3 DIV) was found to significantly reduce LC3B-II levels consistent with mTORC1-mediated autophagy inhibition (Fig. 7B). The ability of T30 to reduce LC3B-II was blocked by co-application of bgtx (p = 0.428) consistent our model of α7 nAChR regulation of autophagy during growth.

 The mTOR pathway coordinates mitochondrial energy production and can regulate the synthesis of various mitochondrial proteins^59^. Our proteomic analysis indicates an effect of T30 on the expression of mitochondrial proteins (Fig. 2 and Table 1). Indeed, AChE and α7 nAChR are individually  reported to regulate mitochondrial activity and contribute to apoptotic signaling in neurons^60,61^. We examined the effect of T30 on cytochrome C levels within SH-SY5Y cells. Immunofluorescence analysis using an anti-cytochrome C antibody shows that T30 treatment reduces cytochrome C expression in cells relative to controls (n = 40, p = 0.009). In these experiments, the inactive portion of T30 (T15) did not have an effect on cytochrome C levels  (Fig. 7C) (n = 40, p = 0.459).

### T30 promotes dendritic growth in hippocampal neurons

**Figure 8:**
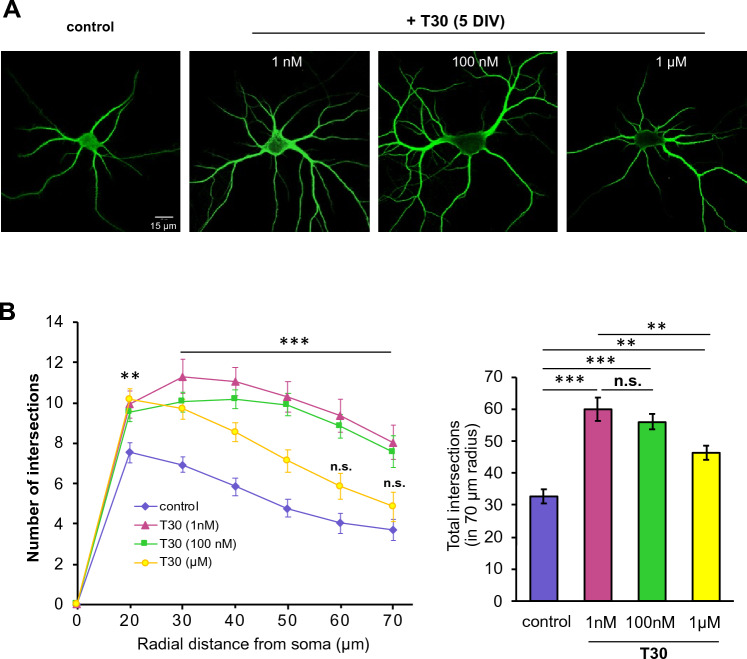
T30 promotes dendritic growth in hippocampal neurons. (**A**) Representative images of anti-MAP2 immunolabeled neurons at 5 DIV of T30 treatment. (**B**) Scholl analysis results showing an effect of T30 (1 nM, 100 nM, 1 μM) on dendritic growth and branching. T30 was found to impact dendritic growth as measured by the number of intersections across concentric shells from the soma. n = 30 cells per group (one-way ANOVA, *p < 0.05, **p < 0.005, ***p < 0.001, Supplemental Table S3).

We confirmed the effect of T30 in primary hippocampal neurons. Previous studies have described acute pharmacological responses of neural cells to T30 along a varied concentration range (2.5 nM to 40 μM)^35^. We tested the impact of T30 on neuronal growth  at 1 nM, 100 nM, and 1 μM corresponding to low, medium, and high non-toxic levels^35^. In these experiments, neurons were treated with various T30 concentrations for 5 DIV and then immunostained with anti-MAP2 antibodies to visualize dendritic arbors (Fig. 8A). A quantitative measure of dendritic length and branching was obtained using a Scholl analysis^34^. As shown in Fig. 8B and Supplemental Table S3, T30 treatment clearly increased dendritic growth relative to controls. A comparison of concentrically defined intersection points at specific distances from the soma, indicates that T30 promotes significant dendritic growth at 1 nM and 100 nM but shows a diminished growth effect at 1 μM concentration. This observation was robust across two forms of morphometric analyses of dendritic growth and underscore dose-related actions of T30 in growth.

## Discussion

AChE is an enzyme vital for mammalian synaptic transmission through its ability to hydrolyze ACh^62,63^. It is also widely expressed outside of the nervous system and is sometimes found in non-cholinergic cells^64^. A large body of work has demonstrated non-hydrolytic properties for AChE within various cell types^12–14^. Amongst its non-hydrolytic activity is its role as a trophic factor during normal development and cancer^65^. Studies show that synaptic AChE-T is especially abundant during brain development and can regulate axonal growth, cell adhesion, as well as pathfinding during synaptogenesis^66,67^. The loss of AChE-T in mice is associated with disruption to synaptic connectivity within the retina and cortex^68^. Our study supports the involvement of non-hydrolytic AChE-T in growth demonstrating an important role for the T30 peptide in human neural cells^69,70^. We add to this body of evidence by showing that T30 can signal growth in human SHY-SY5Y cells and primary hippocampal neurons. In neurons, we find that T30 activates dendritic growth within a specific concentration range, consistent with the idea that T30 allosteric ligand site within the ⍺7 nAChR^35^.

Earlier findings demonstrate that T30 binds the ⍺7 nAChR^48^. Sequence similarity between T30 and Aβ42 has been shown yet it is not yet clear if the two peptides share common binding partners in cells. Studies have shown that brain AChE complexes with Aβ peptide fragments^71^, and that AChE and butyrylcholinesterase are present within Aβ deposits such as senile plaque^72^. Interestingly, in ex vivo rat brain slices, the application of T30 results in an increase in the expression of Aβ42^73^, suggesting that T14 interferes with amyloid protein turnover. Interactions between AChE and nAChR have also been explored in other contexts^74^. Thus, while the two molecules are co-expressed at the mature cholinergic synapse, AChE-T and the ⍺7 nAChR appear highly coupled in expression during brain development^75^. In early post-natal synaptic development, α7 nAChR is at its highest within rodent brain and shown to regulate neural cell proliferation and synaptic maturation within regions such as the hippocampus^10^. Our earlier studies have shown an important role for α7 nAChR metabotropic signaling through G proteins in axonal calcium signaling and growth cone motility^29^. In this study, T30 activation of the α7 nAChR is also able to support neurite growth through a process that appears dependent on G protein signaling since expression of the α7_345–348A_ mutant did not support  T30 mediated growth.

Our proteomic analysis reveals several important intracellular pathways engaged by the presentation of T30 in vitro. These pathways all appear to promote cell growth and protein synthesis. In fact, when looking at statistically altered proteins within the T30 proteome, ~ 75% of the change was due to an increase in the expression of proteins. Bioinformatic analysis using MCL and DAVID KEGG pathway indicates that these protein changes reflect an mTOR pro-growth state within treated cells. mTOR is an evolutionarily conserved serine/threonine kinase that regulates many cellular responses (from autophagy to translation) and organizes molecular responses to incoming signals by modulating energy and metabolic substrates^76,77^. It functions via two distinct complexes: mTORC1 and mTORC2 with the activation mTORC1 resulting in protein synthesis via p70 S6 kinase (S6K1 and S6K2) and phosphorylation of eukaryotic initiation factor 4-binding protein (4EBP1 and 4EBP2). mTORC1 also suppresses autophagy mediated protein degradation and can contribute to growth^56^. Our findings show that T30 promotes neural cell growth by activating α7 nAChRs leading to mTOR pathway signaling. This process is supported by recently published evidence on the ability of α7 nAChRs to direct AKT/mTOR autophagy within neurons^78,79^.

Our experiments show that T30 acts via mTOR at several points, first by decreasing the expression of the autophagy marker LC3B and second by increasing the phosphorylation of both eIF4E and S6K. These processes appear to promote neurite growth and explain the actions of T30 on growth within our cells as well as elsewhere^54,56^. Interestingly, the effects of T30 appear accompanied by overall reduction in cellular cytochrome c, which is a driver of apoptosis^80^. The effects of T30 are found specific since antagonism of the α7 nAChR with bgtx abolished T30-associated growth, and application of the mTOR inhibitor rapamycin and non-bioactive peptide variants of T30 (T15 and NBP14) did not promote growth signaling. Studies have shown interactions between nAChRs and intracellular organelle including the ER and mitochondria^9,19^. In future studies, it will be important to examine subcellular compartment specific proteomic changes that accompany T30 activation of the nAChR.

Disruption to mTOR signaling is implicated in many human disease including auto-immune disorder, neurodegeneration, and various cancers^50^. In the brain stimulation of mTOR has been suggested to promote hyperphosphorylation of synaptic tau and can drive amyloid protein accumulation^81^. A growing body of evidence demonstrates a link between mTOR signaling and AD. For example, an alteration in the autophagy-lysosome pathway has been shown to drive Aβ42 neurotoxicity^82,83^, and a loss in mTORC1 regulation appears to contribute to protein aggregation within neural cells^84^. It has been suggested that interactions between T30 and nAChRs can participate in early cholinergic cell death within the brain^85^. This study provides novel evidence on a connection between the mTOR pathway and ⍺7 nAChR signaling. How this may contribute to neural development and disease is an important question for the future studies.

## Supplementary Information

Supplementary Information.

## Data Availability

Proteomic data generated during this study is deposited in the online open access Figshare repository (https://doi.org/10.6084/m9.figshare.22637611.v1).
