# Supplementary material for: The human acetylcholinesterase C-terminal T30 peptide activates neuronal growth through alpha 7 nicotinic acetylcholine receptors and the mTOR pathway
Source: Sci Rep. 2023 Jul 15;13:11434. doi: 10.1038/s41598-023-38637-1 (PMC10349870; doi:10.1038/s41598-023-38637-1)

SUPPLEMENTARY MATERIAL “The human acetylcholinesterase c-terminal T30 peptide activates neuronal growth through alpha 7 nicotinic acetylcholine receptor and the mTOR pathway”, Graur et al., 2023

Supplemental Table 1: Peptide sequences.

Supplemental Table 2: A complete list of significantly altered proteins and within SH-SY5Y cell treated with 100nM T30 for 3 DIV.

Supplemental Table 3: ANOVA and Tukey HSD / Tukey Kramer output for Sholl analysis

Supplement Fig 1. Western blot analysis of Immunoreactivity to elF4E, phospho-elF4E, LC3B.

Supplement Fig 2. Western blot analysis showing immunoreactivity to anti-S6K and tnti-phospho-S6K antibodies.

Supplement Fig 3. Western blot analysis showing immunoreactivity to anti-GAPDH.

Peptide name	Peptide Sequence
T30	KAEFHRWSSYMVHWKNQFDHYSKQDRCSDL
NBP14	AEFHRWSSYMVHWK
T15	NQFDHYSKQDRCSDL

Supplemental Table 1: Peptide sequences.

Supplemental Table 2: A complete list of significantly altered proteins and within SH-SY5Y cell treated with 100nM T30 for 3 DIV. Peptide abundance determined by precursor ion quantification in Proteome Discoverer v2.4.

Gene Symbol	Description	$\log_2$ fold change T30	fold change T30 p-value
A2M	alpha-2-macroglobulin isoform X1	-2.41E+00	1.02E-07
AKT3	RAC-gamma serine/threonine-protein kinase isoform X1	7.74E-01	3.72E-02
ALB	serum albumin preproprotein	-1.19E+00	4.18E-03

ANKRD44	serine/threonine-protein phosphatase 6 regulatory ankyrin repeat subunit B isoform X1	1.68E+00	3.46E-03
ARF5	ADP-ribosylation factor 5	8.42E-01	6.39E-05
ARHGAP33	rho GTPase-activating protein 33 isoform X9	-1.99E+00	3.22E-03
ARL8A	ADP-ribosylation factor-like protein 8A isoform 1	7.03E-01	6.96E-04
ARMC6	armadillo repeat-containing protein 6 isoform 1	8.75E-01	8.40E-03
ATP2B1	plasma membrane calcium-transporting ATPase 1 isoform X1	1.35E+00	3.76E-04
ATP5IF1	ATPase inhibitor, mitochondrial isoform 1 precursor	-2.23E+00	2.16E-04
ATP6V1C2	V-type proton ATPase subunit C 2 isoform X1	-9.05E-01	8.44E-03
ATP6V1G1	V-type proton ATPase subunit G 1	7.43E-01	2.71E-02
BAG2	BAG family molecular chaperone regulator 2	1.50E+00	4.07E-07
BCAP31	B-cell receptor-associated protein 31 isoform a	-1.46E+00	3.15E-02
BCL2L13	bcl-2-like protein 13 isoform X1	1.20E+00	7.46E-05
BTF3L4	transcription factor BTF3 homolog 4 isoform 1	6.85E-01	1.06E-03
CAMK1G	calcium/calmodulin-dependent protein kinase type 1G	9.85E-01	2.33E-07
CENPF	centromere protein F	1.50E+00	3.46E-02
CHGB	secretogranin-1 precursor	7.98E-01	3.58E-02
CISD3	CDGSH iron-sulfur domain-containing protein 3, mitochondrial precursor	1.24E+00	1.65E-05
CKS2	cyclin-dependent kinases regulatory subunit 2	2.28E+00	5.11E-10
CLUH	clustered mitochondria protein homolog isoform X1	1.03E+00	1.99E-07
COPG1	coatomer subunit gamma-1	7.27E-01	3.05E-02
COTL1	coactosin-like protein	1.44E+00	4.69E-02
COX5A	cytochrome c oxidase subunit 5A, mitochondrial precursor	-9.86E-01	6.22E-03
CRADD	death domain-containing protein CRADD isoform 1	8.00E+00	1.36E-02
CUTA	protein CutA isoform 1	5.68E-01	8.34E-03
DENND2A	DENN domain-containing protein 2A isoform a	1.21E+00	2.56E-03

DEPDC1	DEP domain-containing protein 1A isoform a	1.23E+00	8.15E-05
DHRS7B	dehydrogenase/reductase SDR family member 7B isoform X1	1.69E+00	5.08E-03
DHTKD1	probable 2-oxoglutarate dehydrogenase E1 component DHKTD1, mitochondrial	2.88E+00	3.07E-16
DLL3	delta-like protein 3 isoform 1 precursor	1.07E+00	7.70E-07
DNAAF5	dynein assembly factor 5, axonemal	7.08E-01	1.81E-02
DNAH10	dynein heavy chain 10, axonemal isoform X1	3.34E+00	3.07E-16
DNAJA4	dnaJ homolog subfamily A member 4 isoform 1	1.26E+00	8.46E-10
DNAJB4	dnaJ homolog subfamily B member 4 isoform a	1.07E+00	1.35E-02
DPF2	zinc finger protein ubi-d4 isoform X1	1.40E+00	3.40E-04
EIF4ENIF1	eukaryotic translation initiation factor 4E transporter isoform X1	1.73E+00	2.26E-03
ENOPH1	enolase-phosphatase E1 isoform 1	6.83E-01	1.06E-03
ENY2	transcription and mRNA export factor ENY2 isoform 1	8.38E-01	1.08E-04
EYS	protein eyes shut homolog isoform 4 precursor	1.87E+00	9.77E-03
FAM213A	redox-regulatory protein FAM213A isoform X1	6.20E-01	2.75E-03
FAM3C	protein FAM3C isoform X1	-1.30E+00	9.81E-03
FARS2	phenylalanine--tRNA ligase, mitochondrial	1.49E+00	1.03E-03
FKBP15	FK506-binding protein 15	1.33E+00	5.59E-04
FLJ10769	ATP-dependent (S)-NAD(P)H-hydrate dehydratase isoform a	1.32E+00	1.04E-03
FLOT2	flotillin-2 isoform X1	1.07E+00	8.91E-03
FSD1L	FSD1-like protein isoform X1	2.15E+00	8.46E-10
FSIP2	fibrous sheath-interacting protein 2	-1.09E+00	2.11E-02
GGA2	ADP-ribosylation factor-binding protein GGA2	1.82E+00	5.18E-03
GNE	bifunctional UDP-N-acetylglucosamine 2-epimerase/N-acetylmannosamine kinase isoform 1	9.47E-01	2.44E-02
GOLGA4	golgin subfamily A member 4 isoform X1	1.76E+00	5.33E-03
GTF2A1	transcription initiation factor IIA subunit 1 isoform 1	1.19E+00	3.01E-03
H2AC6	histone H2A type 1-C	6.43E-01	2.92E-03
H3C1	histone H3.1	-1.01E+00	1.31E-03

HAUS8	HAUS augmin-like complex subunit 8 isoform a	1.71E+00	4.00E-03
HBS1L	HBS1-like protein isoform 1	1.46E+00	1.66E-03
HECTD4	probable E3 ubiquitin-protein ligase HECTD4	1.07E+00	7.07E-03
HEXA	beta-hexosaminidase subunit alpha isoform 1 precursor	1.54E+00	3.11E-09
HMCN1	hemicentin-1 precursor	2.58E+00	3.07E-16
HMGN2	non-histone chromosomal protein HMG-17	-1.63E+00	1.83E-02
HNRNPR	heterogeneous nuclear ribonucleoprotein R isoform X1	2.53E+00	3.85E-11
IQSEC1	IQ motif and SEC7 domain-containing protein 1 isoform X2	1.39E+00	1.34E-03
IRAK1	interleukin-1 receptor-associated kinase 1 isoform 1	2.50E+00	2.76E-05
IRF3	interferon regulatory factor 3 isoform X2	1.89E+00	3.51E-04
ISOC2	isochorismatase domain-containing protein 2 isoform 2	9.44E-01	7.24E-03
ISY1	ISY1-RAB43 protein	9.72E-01	4.11E-04
KBTBD3	kelch repeat and BTB domain-containing protein 3 isoform 1	-1.09E+00	4.35E-02
KCNAB2	voltage-gated potassium channel subunit beta-2 isoform X5	1.96E+00	1.89E-03
KHDRBS1	KH domain-containing, RNA-binding, signal transduction-associated protein 1 isoform 1	-1.18E+00	2.48E-04
KIF15	kinesin-like protein KIF15 isoform X1	1.12E+00	1.72E-02
KIF2C	kinesin-like protein KIF2C isoform 1	3.30E+00	3.07E-16
LAMB1	laminin subunit beta-1 isoform X1	1.66E+00	1.47E-05
LAMTOR3	ragulator complex protein LAMTOR3 isoform 1	5.50E-01	4.50E-02
LMAN2	vesicular integral-membrane protein VIP36 precursor	-8.97E-01	1.17E-02
LSM8	LSM8 homolog, U6 small nuclear RNA associated	-1.10E+00	4.70E-03
LTN1	E3 ubiquitin-protein ligase listerin isoform 1	1.23E+00	2.55E-03
MAPK1	mitogen-activated protein kinase 1	-1.07E+00	1.88E-02
MCUR1	mitochondrial calcium uniporter regulator 1	-8.60E-01	3.46E-02
METTL16	U6 small nuclear RNA (adenine-(43)-N(6))-methyltransferase	1.64E+00	1.18E-02
METTL3	N6-adenosine-methyltransferase catalytic subunit	5.18E-01	7.73E-03
MRPL50	39S ribosomal protein L50, mitochondrial	6.33E-01	3.22E-03

MRPS35	28S ribosomal protein S35, mitochondrial isoform 1 precursor	9.77E-01	7.20E-05
MSI2	RNA-binding protein Musashi homolog 2 isoform X1	1.10E+00	9.06E-08
NAGA	alpha-N-acetylgalactosaminidase isoform X1	2.22E+00	2.40E-04
NCOR1	nuclear receptor corepressor 1 isoform X1	7.71E-01	2.01E-02
NDE1	nuclear distribution protein nudE homolog 1 isoform X5	1.42E+00	3.84E-04
NDUFAF4	NADH dehydrogenase [ubiquinone] 1 alpha subcomplex assembly factor 4	2.32E+00	6.08E-11
NEDD8	NEDD8 precursor	7.11E-01	2.14E-04
NF1	neurofibromin isoform 1	-1.27E+00	1.50E-02
NME1	nucleoside diphosphate kinase A isoform a	-1.09E+00	4.17E-02
NMRAL1	nmrA-like family domain-containing protein 1 isoform X2	1.64E+00	3.07E-16
NMU	neuromedin-U isoform 1 preproprotein	1.67E+00	7.95E-03
NOC3L	nucleolar complex protein 3 homolog	7.92E-01	1.38E-03
NOL8	nucleolar protein 8 isoform X1	9.15E-01	4.83E-02
NOSIP	nitric oxide synthase-interacting protein isoform X3	7.65E-01	2.36E-02
NT5DC1	5'-nucleotidase domain-containing protein 1	1.03E+00	2.38E-02
NUCB1	nucleobindin-1 isoform X1	4.09E-01	3.94E-02
NVL	nuclear valosin-containing protein-like isoform X2	2.41E+00	1.56E-04
NXN	nucleoredoxin isoform 1	1.37E+00	1.41E-02
OPA1	dynamin-like 120 kDa protein, mitochondrial isoform 8	5.50E-01	1.62E-02
ORC2	origin recognition complex subunit 2 isoform X1	2.00E+00	4.11E-04
PLRG1	pleiotropic regulator 1 isoform 1	-9.49E-01	2.84E-02
PODXL2	podocalyxin-like protein 2 precursor	1.98E+00	1.31E-03
PPFIA1	liprin-alpha-1 isoform X8	9.99E-01	6.38E-04
PTMA	prothymosin alpha isoform X1	-1.46E+00	4.98E-02
PTPN2	tyrosine-protein phosphatase non-receptor type 2 isoform X1	1.87E+00	2.75E-10
PUS1	tRNA pseudouridine synthase A isoform 1	1.52E+00	9.33E-05
RAB11FIP1	rab11 family-interacting protein 1 isoform 3	1.21E+00	9.66E-03
RFC2	replication factor C subunit 2 isoform 1	8.10E-01	1.96E-02
RIPK1	receptor-interacting serine/threonine-protein kinase 1 isoform 1	1.50E+00	3.36E-02

RPL15	60S ribosomal protein L15 isoform 1	-8.62E-01	1.99E-02
RPL26L1	60S ribosomal protein L26-like 1	-1.70E+00	3.07E-02
RPLP1	60S acidic ribosomal protein P1 isoform 1	5.70E-01	3.13E-03
RPS27	40S ribosomal protein S27 isoform 1	-1.09E+00	4.92E-03
RPS27A	ubiquitin-40S ribosomal protein S27a precursor	4.93E-01	1.19E-02
RPS5	40S ribosomal protein S5	-8.78E-01	1.54E-02
RSBN1L	round spermatid basic protein 1-like protein	1.07E+00	1.62E-02
RSU1	ras suppressor protein 1 isoform 1	1.62E+00	1.44E-05
S100A6	protein S100-A6	1.13E+00	4.09E-03
SCAF4	splicing factor, arginine/serine-rich 15 isoform 1	-1.29E+00	2.23E-03
SCOC	short coiled-coil protein isoform 1	1.40E+00	3.51E-04
SEC22B	vesicle-trafficking protein SEC22b precursor	5.18E-01	2.70E-02
SETD3	histone-lysine N-methyltransferase setd3 isoform X1	1.17E+00	3.36E-03
SGF29	SAGA-associated factor 29	2.03E+00	1.12E-08
SLC12A9	solute carrier family 12 member 9 isoform X1	1.80E+00	1.31E-03
SPG7	paraplegin isoform X1	2.67E+00	3.07E-16
SPIRE1	protein spire homolog 1 isoform X1	1.73E+00	2.36E-02
SRSF11	serine/arginine-rich splicing factor 11 isoform 3	4.83E-01	3.21E-02
STARD7	stAR-related lipid transfer protein 7, mitochondrial precursor	1.30E+00	2.53E-05
STK38	serine/threonine-protein kinase 38 isoform X1	5.33E-01	1.45E-02
STUB1	E3 ubiquitin-protein ligase CHIP isoform a	5.15E-01	1.45E-02
SUMO2	small ubiquitin-related modifier 2 isoform a precursor	-1.16E+00	1.23E-04
TAF1	transcription initiation factor TFIID subunit 1 isoform X1	1.15E+00	7.02E-04
TECPR1	tectonin beta-propeller repeat-containing protein 1	1.39E+00	4.86E-02
THOC2	THO complex subunit 2 isoform X1	1.20E+00	2.00E-03
TLR7	toll-like receptor 7 precursor	-1.91E+00	2.97E-03
TMA7	translation machinery-associated protein 7 isoform 1	-1.58E+00	7.11E-05
TMEM167A	protein kish-A precursor	1.42E+00	4.56E-02
TMEM230	transmembrane protein 230 isoform X1	-1.47E+00	4.36E-02

TMSB10	thymosin beta-10	-1.31E+00	1.04E-03
TOMM40	mitochondrial import receptor subunit TOM40 homolog	1.32E+00	4.79E-02
TP53BP1	TP53-binding protein 1 isoform 1	2.77E+00	9.04E-07
TP53RK	TP53-regulating kinase	1.48E+00	3.85E-02
TPM3	tropomyosin alpha-3 chain isoform Tpm3.2cy	2.26E+00	3.07E-16
TXN2	thioredoxin, mitochondrial isoform X1	1.92E+00	5.16E-04
UBE2A	ubiquitin-conjugating enzyme E2 A isoform 1	-1.50E+00	4.23E-02
UBE3C	ubiquitin-protein ligase E3C	6.67E-01	2.65E-02
UQCRFS1	cytochrome b-c1 complex subunit Rieske, mitochondrial	1.24E+00	6.33E-05
USP9X	probable ubiquitin carboxyl-terminal hydrolase FAF-X isoform X1	5.55E-01	2.50E-02
UTP14A	U3 small nucleolar RNA-associated protein 14 homolog A isoform 1	1.68E+00	1.41E-02
WDR3	WD repeat-containing protein 3	2.27E+00	5.77E-04
XPNPEP3	probable Xaa-Pro aminopeptidase 3 isoform 1	-8.65E-01	1.85E-02
YBX1	nuclease-sensitive element-binding protein 1	-8.55E-01	3.63E-02
ZDHHC6	palmitoyltransferase ZDHHC6 isoform X6	-2.44E+00	1.39E-04
ZNF48	zinc finger protein 48 isoform 1	1.29E+00	3.31E-03
ZNF830	zinc finger protein 830	1.63E+00	1.61E-02

Supplemental Table 3: One-Way ANOVA and Tukey HSD / Tukey Kramer output for Sholl analysis

Radius (μm)	One-Way ANOVA p-value
20	0.00424426
30	5.87863E-06
40	5.37E-10
50	3.50E-10
60	3.29E-08
70	5.01569E-05

Tukey HSD / Tukey Kramer test result at the 20 μM radius:

Pair	Difference	SE	Q	Lower CI	Upper CI	Critical Mean	p-value
Control vs 1nM	2.4	0.5579	4.3022	0.3436	4.4564	2.0564	0.0152
Control vs 100nM	2	0.5579	3.5852	-0.05645	4.0564	2.0564	0.0598

Control vs 1µM	2.6333	0.5579	4.7205	0.5769	4.6898	2.0564	0.006163
1nM vs 100nM	0.4	0.5579	0.717	-1.6564	2.4564	2.0564	0.9573
1nM vs 1µM	0.2333	0.5579	0.4183	-1.8231	2.2898	2.0564	0.991
100nM vs 1µM	0.6333	0.5579	1.1353	-1.4231	2.6898	2.0564	0.853

Tukey HSD / Tukey Kramer test result at the 30 µM radius:

Pair	Difference	SE	Q	Lower CI	Upper CI	Critical Mean	p-value
Control vs 1nM	4.3667	0.5792	7.5392	2.2315	6.5018	2.1351	2.9E-06
Control vs 100nM	3.1	0.5792	5.3522	0.9649	5.2351	2.1351	0.001385
Control vs 1µM	2.7667	0.5792	4.7767	0.6315	4.9018	2.1351	0.00543
1nM vs 100nM	1.2667	0.5792	2.1869	-0.8685	3.4018	2.1351	0.4134
1nM vs 1µM	1.6	0.5792	2.7624	-0.5351	3.7351	2.1351	0.2119
100nM vs 1µM	0.3333	0.5792	0.5755	-1.8018	2.4685	2.1351	0.9771

Tukey HSD / Tukey Kramer test result at the 40 µM radius:

Pair	Difference	SE	Q	Lower CI	Upper CI	Critical Mean	p-value
Control vs 1nM	5.2	0.5289	9.8326	3.2505	7.1495	1.9495	1.41E-09
Control vs 100nM	4.3667	0.5289	8.2569	2.4171	6.3162	1.9495	2.92E-07
Control vs 1µM	2.7333	0.5289	5.1684	0.7838	4.6829	1.9495	0.002171
1nM vs 100nM	0.8333	0.5289	1.5757	-1.1162	2.7829	1.9495	0.6815
1nM vs 1µM	2.4667	0.5289	4.6642	0.5171	4.4162	1.9495	0.006988
100nM vs 1µM	1.6333	0.5289	3.0885	-0.3162	3.5829	1.9495	0.1338

Tukey HSD / Tukey Kramer test result at the 50 µM radius:

Pair	Difference	SE	Q	Lower CI	Upper CI	Critical Mean	p-value
Control vs 1nM	5.5333	0.5906	9.3691	3.3562	7.7105	2.1772	6.90E-09
Control vs 100nM	5.1333	0.5906	8.6918	2.9562	7.3105	2.1772	6.91E-08
Control vs 1µM	2.3667	0.5906	4.0073	0.1895	4.5438	2.1772	0.02746
1nM vs 100nM	0.4	0.5906	0.6773	-1.7772	2.5772	2.1772	0.9636
1nM vs 1µM	3.1667	0.5906	5.3618	0.9895	5.3438	2.1772	0.001352
100nM vs 1µM	2.7667	0.5906	4.6845	0.5895	4.9438	2.1772	0.006679

Tukey HSD / Tukey Kramer test result at the 60 µM radius:

Pair	Difference	SE	Q	Lower CI	Upper CI	Critical Mean	p-value
Control vs 1nM	5.3333	0.6556	8.1355	2.9167	7.75	2.4166	4.34E-07
Control vs 100nM	4.8	0.6556	7.322	2.3834	7.2166	2.4166	5.67E-06
Control vs 1µM	1.8333	0.6556	2.7966	-0.5833	4.25	2.4166	0.2025

1nM vs 100nM	0.5333	0.6556	0.8136	-1.8833	2.95	2.4166	0.9393
1nM vs 1μM	3.5	0.6556	5.3389	1.0834	5.9166	2.4166	0.001431
100nM vs 1μM	2.9667	0.6556	4.5254	0.55	5.3833	2.4166	0.009474

Tukey HSD / Tukey Kramer test result at the 70 μM radius:

Pair	Difference	SE	Q	Lower CI	Upper CI	Critical Mean	p-value
Control vs 1nM	4.3333	0.7287	5.9463	1.6469	7.0197	2.6864	2.99E-04
Control vs 100nM	3.8667	0.7287	5.306	1.1803	6.5531	2.6864	1.55E-03
Control vs 1μM	1.1667	0.7287	1.6009	-1.5197	3.8531	2.6864	0.6706
1nM vs 100nM	0.4667	0.7287	0.6404	-2.2198	3.1531	2.6864	0.969
1nM vs 1μM	3.1667	0.7287	4.3454	0.4802	5.8531	2.6864	0.01389
100nM vs 1μM	2.7	0.7287	3.705	0.01358	5.3864	2.6864	0.04835

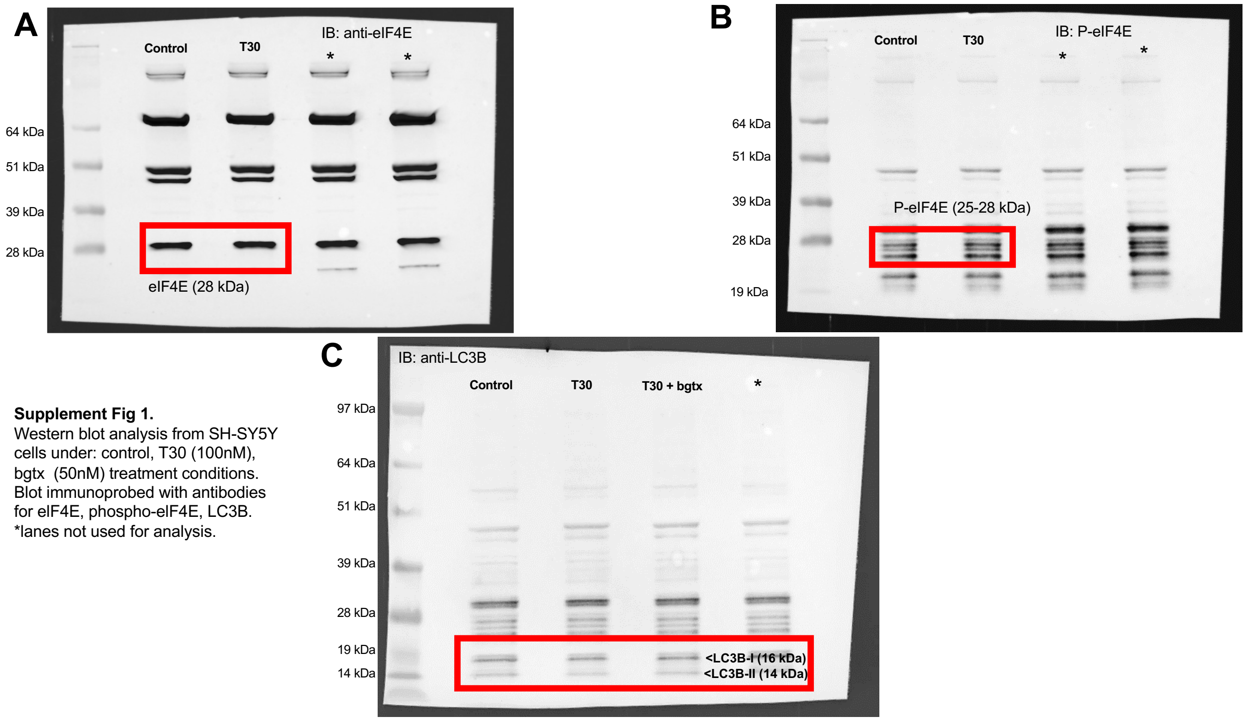

Supplement Fig 1.
Western blot analysis from SH-SY5Y cells under: control, T30 (100nM), bgtx (50nM) treatment conditions. Blot immunoprobed with antibodies for eIF4E, phospho-eIF4E, LC3B.
*lanes not used for analysis.

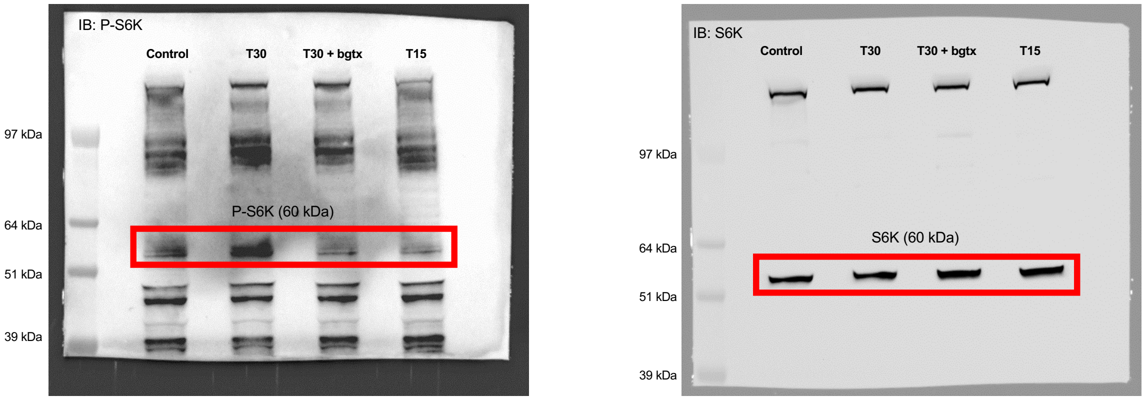

Supplement Fig 2.
Western blot analysis showing immunoreactivity to anti-S6K and tnti-phospho-S6K antibodies. Lanes loaded with lysates from SH-SY5Y cells under control, T30 (100nM), Bgtx (50nM), T15 (100nM) treatment conditions.

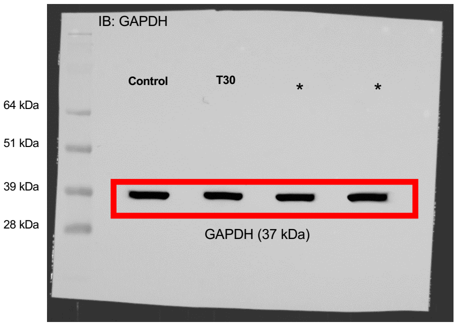

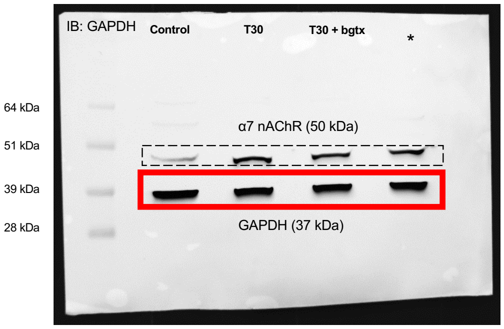

Supplement Fig 3.
Western blot analysis showing immunoreactivity to anti-GAPDH. Lanes are loaded with lysates of SH-SY5Y cells treated with: control, T30 (100nM), Bgtx (50nM) treated conditions.
*lanes not used for analysis.

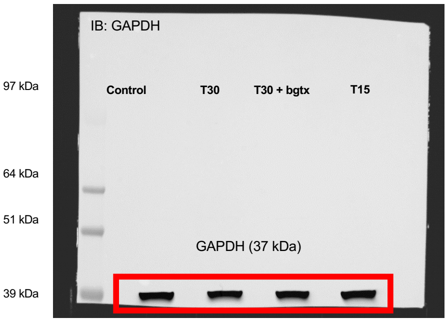

Supplement: Supplementary file 1 — Supplementary Information. [file 41598_2023_38637_MOESM1_ESM.pdf]
